# Diagnosis and surgical management of malignant ovarian teratoma in a green iguana (*Iguana iguana*)

**DOI:** 10.1186/s12917-016-0773-x

**Published:** 2016-07-19

**Authors:** Lucia Bel, Marco Tecilla, Gabriel Borza, Cosmin Pestean, Robert Purdoiu, Ciprian Ober, Liviu Oana, Marian Taulescu

**Affiliations:** Department of Surgery, University of Agricultural Sciences and Veterinary Medicine, 3-5 Mănăştur Street, Cluj-Napoca, 400372 Romania; Department of Veterinary Sciences and Public Health, University of Milan, Milan, Italy; Department of Veterinary Pathology, University of Agricultural Sciences and Veterinary Medicine, 3-5 Mănăştur Street, Cluj-Napoca, 400372 Romania; Department of Anesthesiology and Intensive Care, University of Agricultural Sciences and Veterinary Medicine, 3-5 Mănăştur Street, Cluj-Napoca, 400372 Romania; Department of Radiology, University of Agricultural Sciences and Veterinary Medicine, 3-5 Mănăştur Street, Cluj-Napoca, 400372 Romania; Department of Surgical Techniques, University of Agricultural Sciences and Veterinary Medicine, 3-5 Mănăştur Street, Cluj-Napoca, 400372 Romania

**Keywords:** Green iguana, Ovarian malignant teratoma, Pathology, Reptile, Surgery

## Abstract

**Background:**

Ovarian tumors in reptiles are uncommonly reported in the literature and for green iguanas previously reported cases include teratomas, one adenocarcinoma and one papillary cystadenocarcinoma. The present report is the first of a malignant ovarian teratoma in a green iguana. Complete and detailed pathological features, differential diagnosis and surgical management of malignant ovarian teratoma are discussed in this paper.

**Case Presentation:**

A 9-year-old intact female green iguana (*Iguana iguana*) with a clinical history of persistent anorexia and progressive abdominal distension was referred to the surgery department. On physical examination, a presumptive diagnosis of follicular stasis was established. Radiographic evaluation showed a large radioopaque mass within the abdomen, which was visible both in latero-lateral and ventro-dorsal exposures. Abdominal ultrasonography showed a large intra-abdominal mass, with numerous cyst-like structures filled with liquid and a heterogeneous aspect with hypoechoic areas. Exploratory laparatomy was thus suggested and the mass was removed surgically. The histologic findings of the neoplasm were consistent with those of ovarian malignant teratoma. Surgical excision of the mass in our case was considered curative and after a follow-up period of 6 months the animal has recovered completely.

**Conclusions:**

A malignant ovarian teratoma has not been previously reported in green iguana and should be included in the list of differential diagnosis of ovarian tumors in this species. This report will contribute to a better understanding of the pathology of this rare tumor in green iguanas.

## Background

In domestic mammals, primary ovarian tumors are classified into 3 different categories based on the embryological cell of origin of the predominating neoplastic cell: epithelial tumors (adenocarcinoma and adenoma), germ cell tumors (dysgerminomas and teratomas), and sex cord tumors (GCT, thecoma, granulosa-theca cell, and luteoma) [1].

Ovarian neoplasms have been reported in different species including reptiles [2–4], but among them malignant teratomas are reported as rare. Previously reported ovarian tumors in green iguanas, include teratomas [5, 6], one adenocarcinoma [7], and one papillary cystadenocarcinoma [4].

Teratoma is a gonadal germ cell tumour that predominantly occurs in the gonads: the testis and ovaries [8]. The tumor is based on primordial germ cells from the top cell layer of the blastocyst. From this arise the ectodermal, mesodermal and endodermal germ cell layers [9]. The content of teratomas is complex, reflecting their heterogeneity of germ-cell origin. Neural tissue, woven bone, hyaline cartilage, hair follicles, sebaceous and apocrine glands, respiratory epithelium and adipose tissue have all been reported in animals [10–12]. These histological elements are either seen in associations that resemble normal organs or intermingled haphazardly [13]. Teratomas are classified as benign (mature) or malignant (immature) depending on the degree of anaplasia or the presence of undifferentiated elements resembling those of the embryo [14]. Moreover, the term teratocarcinoma is used only for malignant tumors, which are malignant by virtue of the continued presence of stem cells-the embryonal carcinoma (EC) cells [15].

The current report is the first of a malignant ovarian teratoma in a green iguana. Pathological features, differential diagnosis and surgical management of malignant ovarian teratoma are also discussed in this paper.

## Case Presentation

**Fig. 1:**
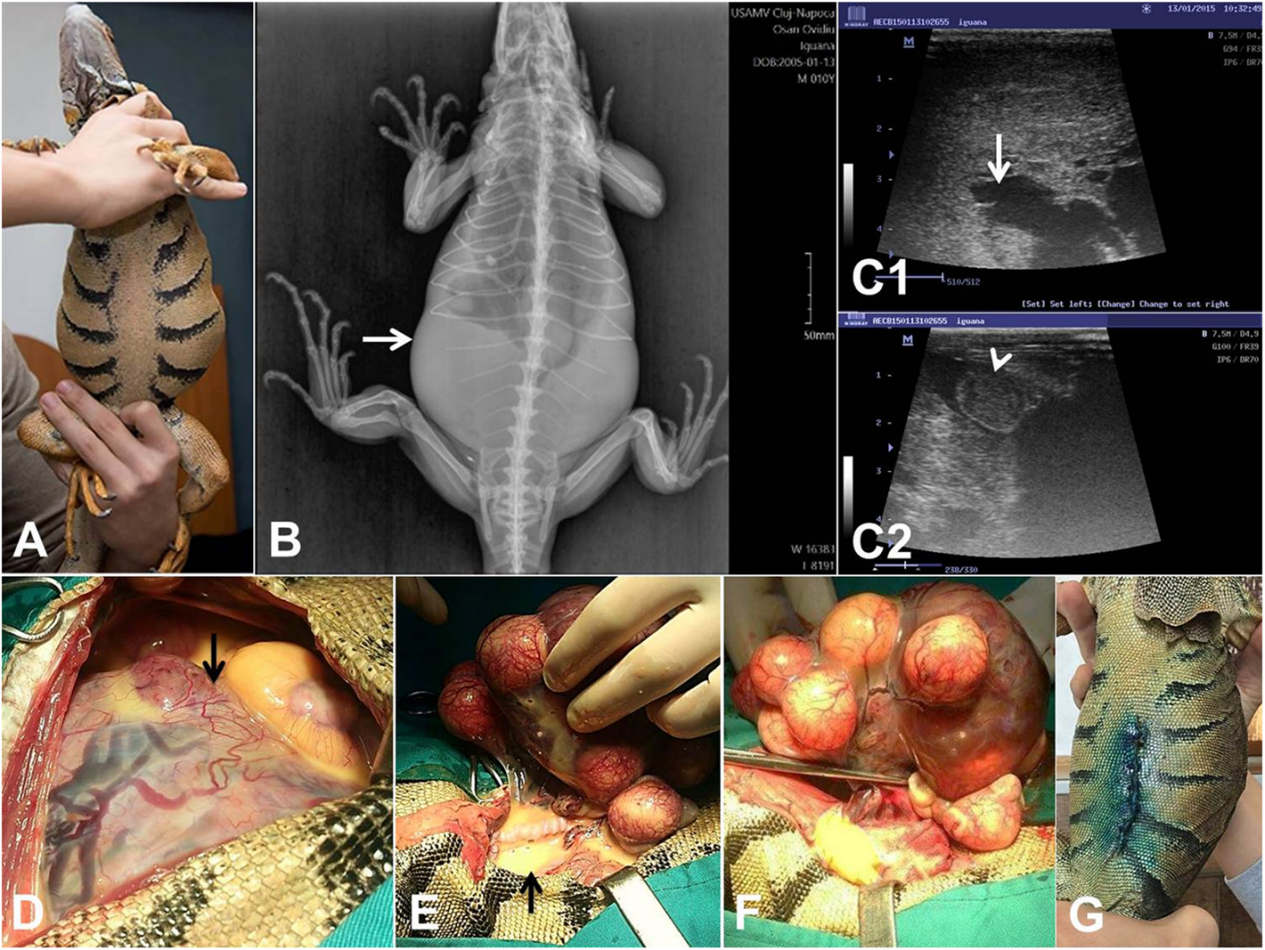
Clinical aspects and surgical management of ovarian teratocarcinoma in Green Iguana. **a** The iguana presenting a marked abdominal distension. **b** Ventro-dorsal radiologic appearance. Note the distended abdomen (arrow). **c1** Ultrasonography showing a large mass inside the abdomen, with cyst like structures filled with liquid. **c2** A round hypoechoic mass of approximately 2.5/1.5 cm surrounded by liquid was identified by ultrasonography. **d** and **e**) Egg yolk content present in the coelomic cavity. **f** Excision of the left ovary. **g** Final aspect of the surgery

In February 2015, a 9 year old Green Iguana (*Iguana iguana*) was presented for consult with a history of 3 weeks anorexia and a distended abdomen (Fig. 1a). Prior to the consult the patient was believed to be a male and was diagnosed with coprostasis. After the initial examination, a presumptive diagnosis of follicular stasis was made, due to the fact that the patient was in fact a female. Blood was collected from the ventral coccygeal vein for hematological and biochemistry evaluation, with no significant alterations. Both a full body radiographs and an abdominal ultrasound were performed. Radiographic evaluation showed a large radiopaque intra-abdominal mass, that was visible both in latero-lateral and ventro-dorsal exposure (Fig. 1b).

The abdominal ultrasound examination was performed using a Mind Ray DC-6 ultrasound device with a linear probe of 7.5–10 MHz. The 7.5 MHz frequency was enough to highlight the abdominal modification. B-Mode abdominal ultrasonography showed a large mass into the coelomic cavity (Fig. 1c1), with numerous cyst-like structures filled with liquid. On ultrasound, the coelomic cavity was partially filled by liquid surrounding an approximatelly 2.5 × 1.5 cm (Fig. 1c2) hypoechoic mass on the lateral right side and fully formed eggs on the left side. Exploratory laparatomy was thus suggested.

Prior to surgery, meloxicam (Metacam®, Boehringer Ingelheim, Germany) at 0.2 mg/kg and butorphanol (Butomidor®, Richter Pharma ag, Austria) at 1 mg/kg were administered in the musculature of the right thoracic limb. After the intravenous induction of anesthesia with alphaxalone (Alfaxan®, Vetoquinol, France) at 15 mg/kg, the animal was intubated using a 3.5 endotracheal tube and was kept on IPPV ventilation, using Isoflurane 1–1.5 % (Anesteran®, Rompharm Company SRL, Romania) and 0.6 l/min air.

The laparatomy was performed using a paramedian craniocaudal incision. Egg yolk content was present in the coelomic cavity (Fig. 1d, e), probably due to the massages that were performed while the animal was presumably coprostatic. After a more thorough examination, a large mass was identified on the left ovary. This mass was then removed, after clamping (Fig. 1f) and ligating the mezovarium vessels with monofilament suture material (Polidioxanone® 3.0, BioSintex, Romania). Ovariectomy of the right ovary was then performed and both the mass and the ovary were submitted for histological analysis. Warm saline lavage was used to remove as much yolk leakage as possible and the abdominal muscles were sutured in a simple interrupted pattern using 3.0 polidioxanone. Skin was closed in a vertical pattern using 3.0 monofilament non absorbable suture material (Nylon®, BioSintex, Romania) (Fig. 1g).

Postoperative, the iguana received 20 mg/kg cephtazidime (Fortum®, GlaxoSmithKline, UK) every 72 h, 10 administrations, 0.2 mg/kg meloxicam every 48 h, 4 administration and oral fluid therapy. Several days after surgery the animal was offered food, but refused to eat, and Emeraid Herbivore® Critical Care was administered. One month after surgery a biochemical recheck was done, using Avian/Reptile profile (Abaxis, Germany) proving no significant alterations and the patient was discharged after the removal of the skin suture. 6 months after surgery, the animal has recovered completely. An abdominal ultrasound was performed, with no evidence of regrowth.

**Fig. 2:**
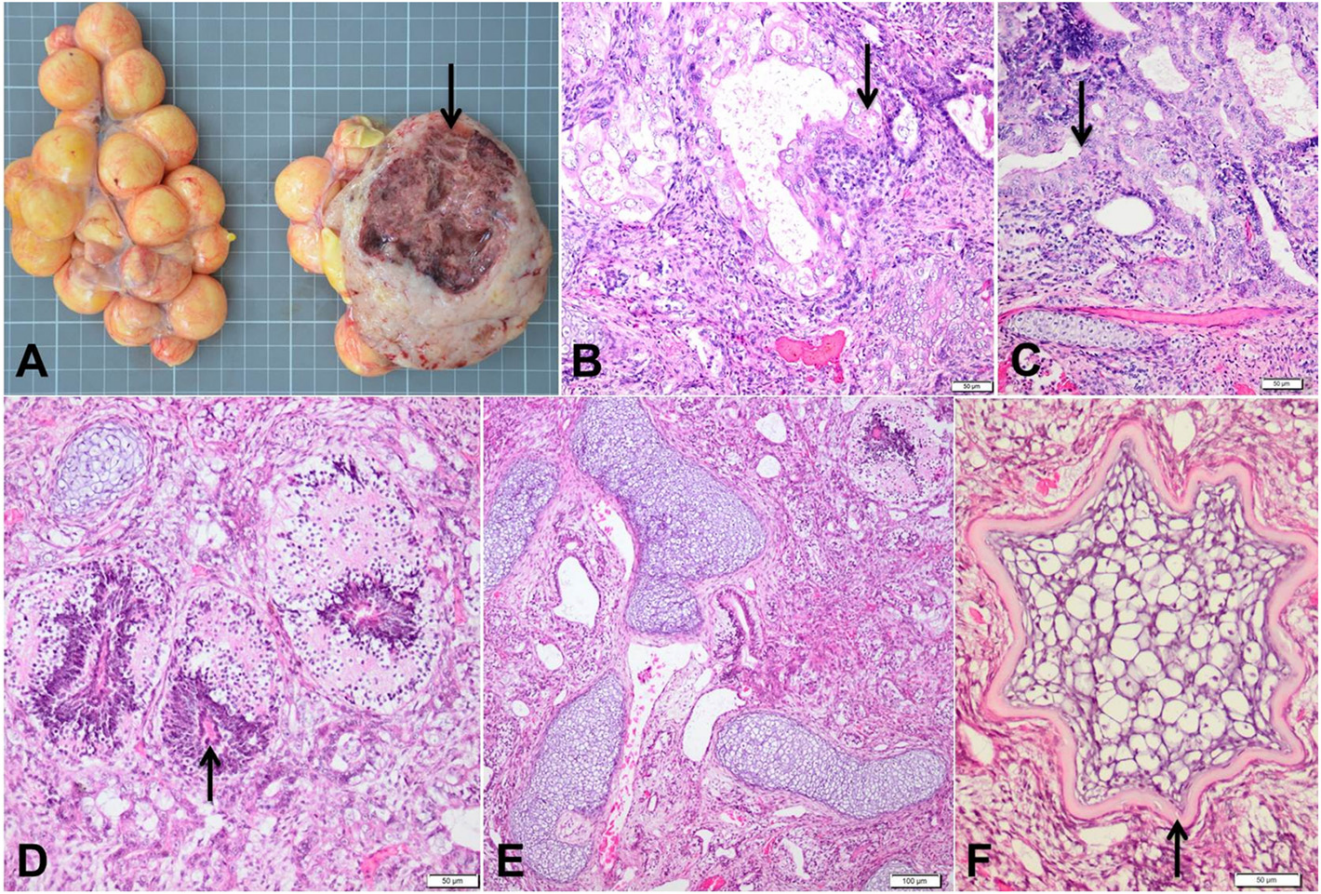
Pathological features of ovarian malignant teratoma in Green Iguana. **a** The neoplastic mass showing multiple necrotic and haemorrhagic areas and variably in size cystic filled cavities containing reddish to brownish fluid. **b** Histologic section of the teratoma showing epithelial cells arranged in cords and islet, multifocally circumscribing variable in size cysts filled with a pale eosinophilic and globous material (proteinaceous material). Hematoxylin and eosin (H&E) stain. Bar = 50μm. **c** Epithelial population organized in acini and tubules lined by 1 to 7 layers of cells. H&E stain. Bar = 50μm. **d** Small and round islet of nervous tissue, characterized by a central channel surrounded by a concentric layer of epithelial cells and abundant neuropil. H&E stain. Bar = 50μm. **e** Neoplastic tissue composed by normal and mature cartilage tissue. H&E stain. Bar = 1000μm. **f** Islets of chondrocytes focally surrounded by a thin layer of mature bone tissue. H&E stain. Bar = 50μm

Grossly, rising from the left ovary, a well-demarcated mass expanding and compressing the surrounding vitellogenic follicles was present. The mass was 9 × 8.5cm in size, with a gray to reddish color and a weight of 340 g compared with the right ovary (203 g). The mass was surrounded by a variably thick, smooth and well vascularized capsule originating from outer layer of the ovary. On section, the neoplastic structure showed multiple necrotic and haemorrhagic areas and variably in size cystic filled cavities containing reddish to brownish fluid (Fig. 2a).

For histological examination, samples from the neoplastic mass were fixed in 10 % phosphate buffered formalin for 24 h, embedded in paraffin wax, cut into 3–5 μm sections, and stained with hematoxylin and eosin.

Histologically, the neoplasm was composed by elements of all germ cell layers (endoderm, mesoderm and ectoderm), haphazardly arranged within the mass. I) Endoderm: Two different epithelial populations were present in the sample. The first one was composed by pleomorphic epithelial cells arranged in cords and islets, multifocally circumscribing variable in size cysts filled with a pale eosinophilic and globous material (proteinaceous material) (Fig. 2b). The cells were cuboidal to polygonal or oval, 30 to 40μm in width, with indistinct cells borders and with an intermediate to high nucleo-cytoplasmic (N/C) ratio. The cytoplasm was moderate in amount, clear and with multiple and variable in size eosinophilic and amorphous granules. The nuclei were large, central to paracentral with a vesicular chromatin and a single nucleolus. Anisokaryosis and anysocytosis were severe with karyomegaly and moderate numbers of mitotic figures. Cytological characteristics were compatible with atypical granulosa cells. Multifocally, admixed to the neoplastic granulosa cells, numerous polygonal cells of 10–12 μm in diameter, with a pale and homogeneous eosinophilic cytoplasm and a small, round and central nucleus were identified (intermediate cells of the follicle wall).

The second epithelial population was organized in acini and tubules lined by 1 to 7 layers of cells (Fig. 2c). The cells were cuboidal to cylindrical, 10–15 μm in diameter, with indistinct cells border and with an intermediate N/C ratio. The cytoplasm was moderate, pale eosinophilic, homogeneous and with an apical brush border. The nuclei were large, round to oval with lacy reticular chromatin. Randomly, a single 2–4 μm in width, eosinophilic nucleolus was detected. Anisokaryosis and anysocytosis were moderate and mitoses were also rare. The lumen of the acini was partially filled with mucus. These epithelial structures could have corresponding to tissue from the respiratory or the genital tract. Multiple small areas of necrosis within the epithelial cell population were identified. II) Ectoderm: A lesser part of the tumor was composed by small and round islet of nervous tissue, characterized by a central channel surrounded by a concentric layer of epithelial cells (ependymal channel) and abundant neuropil (Fig. 2d). III) Mesoderm: Remaining neoplastic tissue was composed by mature hyaline cartilage (Fig. 2e), organized in variably in size islets of chondrocytes focally surrounded by a thin layer of mature bone (Fig. 2f) and scattered foci of striated muscle tissue. All mesenchymal tissues were well differentiated.

According to the largest retrospective publication to date regarding the prevalence of neoplasia in reptiles [16], the tumors are most frequently in snakes, followed by lizards, chelonians, and crocodilians.

Although most gonadal [6] and extragonadal teratomas [12] from animals are benign, malignant teratomas have also been recorded [17]. Histologically, malignant teratomas contain less well-differentiated embryonal elements in addition to mature structures, increased cellular atypia [13] and numerous mitotic figures [17]. Multicentric growth secondary to direct implantation or distant metastases represent other features of malignant teratoma [18]. In the present case, only the endodermal layer showed characteristic features of malignancy characterized by cellular atypia, anysokariosis, karyomegaly, mitoses and necrosis.

Yolk coeliomitis may be the result of yolks being released from the reproductive tract into coelomic cavity or rupture of follicles while still on the ovary [19]. In our case, yolk coeliomitis was caused by rupture of the follicles due to the pressure put on the abdomen while there was a coprostasis suspition.

Iguana ovarian tissue is diffuse and intimately associated with the vena cava and adrenal gland and this makes oophorectomy technically challenging. If the procedure is incomplete, even small remnants will regrow and folliculogenesis will develop [3]. The use of hemostatic clips and microsurgical instruments in complete ovarian removal in reptiles [20] is well known.

In this reported case the bilateral oophorectomy was performed using microsurgical instruments and monofilament absorbable suture material.

Six months later, the patient has recovered completely with no signs of ovarian regrowth.

## Conclusions

To our knowledge, this is the first reported case of malignant ovarian teratoma described antemortem in green iguana (*Iguana iguana*). In our opinion the condition should be included in the list of differential diagnosis of ovarian and other intra-abdominal tumors in this species.

## Abbreviations

GCT, granulosa cell tumor
